# Supplementary material for: Salt-Containing Recipes in Popular Magazines with the Highest Circulation in the United States Do Not Specify Iodized Salt in the Ingredient List
Source: Int J Environ Res Public Health. 2023 Mar 5;20(5):4595. doi: 10.3390/ijerph20054595 (PMC10002291; doi:10.3390/ijerph20054595)
Supplement: Supplementary file 1 [file ijerph-20-04595-s001.zip › ijerph-2203216-supplementary.pdf]

International Journal of
Environmental Research and Public Health

MDPI

Short Communication

Salt-Containing Recipes in Popular Magazines with the Highest Circulation in the United States Do Not Specify Iodized Salt in the Ingredient List

Josephine Uerling [1], Emily Nieckula [2], Katarina Mico [1], Arantxa Bonifaz Rosas [1], Emmie Cohen [1] and Helena Pachón [1,2,*]

Supplementary Materials: Table S1: Recipes reviewed in this study.

Name of magazine	Issue date	Recipe number	Recipe name	Recipe contains salt?	How is salt described?
Taste of Home	June /July 2021	407	Zucchini pickles	Yes	Canning salt
Better Homes & Gardens	September 2021	119	Crispy dill pickled chicken sandwiches	Yes	Celery salt or garlic salt
Better Homes & Gardens	February 2021	603	Cayenne pepper mocktail	Yes	Coarse Kosher salt
Taste of Home	October/November 2020	190	Salty dog sangria	Yes	Coarse sea salt
People	4 October 2021	637	Carne asada jackfruit tacos	Yes	Fine sea salt
Better Homes & Gardens	August 2021	105	Citrus-ginger sports tonic	Yes	Fine Sea salt
Better Homes & Gardens	February 2021	624	Grapefruit & white chocolate cookies	Yes	Fine sea salt
Good Housekeepin g	October 2020	684	Caramel apple pie: for caramel	Yes	Flaked sea salt
Cosmopolitan	March 2021	985	The most non-boring chicken recipe you'll make maybe ever	Yes	Flaky sea salt
People	30 August 2021	643	Mixed tomato caprese salad with balsamic glaze	Yes	Flaky sea salt
Better Homes & Gardens	March 2021	7	Salted pistachio meringues	Yes	Fleur de sel
Taste of Home	August/September 2020	155	Nacho popcorn	Yes	Garlic Salt

Taste of Home	October/November 2020	175	Air-fryer almond chicken	Yes	Garlic Salt
US Weekly	18 October 2021	1001	Cream of tomato soup	Yes	Himalayan sea salt
Taste of Home	February/March 2021	320	Paçoca (Brazilian peanut candy)	Yes	Himalayan sea salt
AARP	February/March 2021	937	Beef and vegetable cocido	Yes	Kosher salt
AARP	August/September 2021		Braised red snapper with lemon & rosemary vinaigrette	Yes	Kosher salt
Cosmopolitan	May/June 2021	953	Nothing to see here, except a secret sauce that'll transform your boring workweek lunches	Yes	Kosher salt
Cosmopolitan	July/August 2021	961	Please, just eat this glorious salad from Pepper Teigen	Yes	Kosher salt
Cosmopolitan	September 2021	966	Cancel your plans and make this sexy breakfast sando	Yes	Kosher salt
Cosmopolitan	October 2020	974	If you've got 25 minutes and a sheet pan, you're a chef now: the food	Yes	Kosher salt
Cosmopolitan	October 2020	975	If you've got 25 minutes and a sheet pan, you're a chef now: the dressing	Yes	Kosher salt
Cosmopolitan	February 2021	991	I'm sorry, you're saying you don't know what a snacking cake is?	Yes	Kosher salt
Good Housekeepin g	September 2021	653	Salted chocolate-caramel cookies	Yes	Kosher salt
Good Housekeepin g	September 2021	655	Chicken paprikash	Yes	Kosher salt
Good Housekeepin g	September 2021	664	Roasted cauliflower tacos	Yes	Kosher salt
Good Housekeepin g	September 2021	665	Quick & spicy vegan slaw	Yes	Kosher salt

Good Housekeeping	September 2021	666	Cheese & herb stuffed chicken	Yes	Kosher salt
Good Housekeeping	September 2021	667	Steak & arugula toasts	Yes	Kosher salt
Good Housekeeping	September 2021	668	Spaghetti & air fryer meatballs	Yes	Kosher salt
Good Housekeeping	September 2021	669	Zucchini bread	Yes	Kosher salt
Good Housekeeping	September 2021	671	Roasted feta shrimp & polenta	Yes	Kosher salt
Good Housekeeping	October 2020	675	Mossy cookies	Yes	Kosher salt
Good Housekeeping	October 2020	680	Chocolate pumpkin bread	Yes	Kosher salt
Good Housekeeping	October 2020	685	Squash & spinach breakfast toasts	Yes	Kosher salt
Good Housekeeping	October 2020	686	Roasted butternut squash salad with tahini vinaigrette	Yes	Kosher salt
Good Housekeeping	October 2020	687	Slow cooker pot roast	Yes	Kosher salt
Good Housekeeping	October 2020	688	Baked rigatoni with meatballs	Yes	Kosher salt
Good Housekeeping	October 2020	690	Brussels sprout pizza	Yes	Kosher salt
Good Housekeeping	November 2020	701	Quinoa-stuffed acorn squash with cranberries and feta	Yes	Kosher salt
Good Housekeeping	November 2020	702	Twice-baked sweet potatoes	Yes	Kosher salt

Good Housekeepin g	November 2020	703	Roasted brussels sprouts with browned butter	Yes	Kosher salt
Good Housekeepin g	November 2020	704	Sheet pan sausage & egg breakfast bake	Yes	Kosher salt
Good Housekeepin g	November 2020	705	Cookie-stuffed brownies	Yes	Kosher salt
Good Housekeepin g	November 2020	709	Herb stuffing: bacon & chestnut	Yes	Kosher salt
Good Housekeepin g	November 2020	710	Herb stuffing: apple, pecan & dill	Yes	Kosher salt
Good Housekeepin g	November 2020	711	Herb stuffing: cornbread, sausage & cherry	Yes	Kosher salt
Good Housekeepin g	November 2020	712	Wild rice & cherry pilaf	Yes	Kosher salt
Good Housekeepin g	November 2020	713	Mushroom & chile-garlic green beans	Yes	Kosher salt
Good Housekeepin g	November 2020	714	Ravioli with sautéed butternut squash & thyme	Yes	Kosher salt
Good Housekeepin g	November 2020	719	Curried rice with shrimp & peas	Yes	Kosher salt
Good Housekeepin g	November 2020	720	Pie-spiced apple fritters	Yes	Kosher salt
Good Housekeepin g	November 2020	726	Chicken with sautéed apples and mushrooms	Yes	Kosher salt
Good Housekeepin g	November 2020	727	Coriander-maple glazed carrots	Yes	Kosher salt
Good Housekeepin g	November 2020	729	Bacon maple-orange turkey	Yes	Kosher salt

Good Housekeeping	November 2020	730	Raspberry swirl rolls	Yes	Kosher salt
Good Housekeeping	November 2020	731	Turkey sandwich	Yes	Kosher salt
Good Housekeeping	May 2021	733	Lemon-thyme chicken	Yes	Kosher salt
Good Housekeeping	May 2021	734	Blackened-fish tacos	Yes	Kosher salt
Good Housekeeping	May 2021	736	Chicken cobb salad	Yes	Kosher salt
Good Housekeeping	May 2021	737	Steak sandwich with grilled broccoli	Yes	Kosher salt
Good Housekeeping	May 2021	738	Lemon bundt cake: for cake	Yes	Kosher salt
Good Housekeeping	May 2021	740	Thin mint cheesecake: for crust	Yes	Kosher salt
Good Housekeeping	May 2021	742	Spice-dusted pork with crunch vegetable salad	Yes	Kosher salt
Good Housekeeping	May 2021	743	Blueberry buttermilk muffins	Yes	Kosher salt
Good Housekeeping	May 2021	744	Crispy chicken with spring quinoa	Yes	Kosher salt
Good Housekeeping	May 2021	746	Cilantro-lime yogurt	Yes	Kosher salt
Good Housekeeping	May 2021	747	Sriracha-honey vinaigrette	Yes	Kosher salt
Good Housekeeping	May 2021	748	Roasted red pepper relish	Yes	Kosher salt

Good Housekeeping	May 2021	750	Buffalo wing burger: for burgers	Yes	Kosher salt
Good Housekeeping	May 2021	751	Celery and blue cheese slaw	Yes	Kosher salt
Good Housekeeping	May 2021	752	Spinach-artichoke deep-dish pizza	Yes	Kosher salt
Good Housekeeping	May 2021	753	Ceviche-style shrimp	Yes	Kosher salt
Good Housekeeping	May 2021	754	Spicy bloody Mary	Yes	Kosher salt
Good Housekeeping	May 2021	756	Herb-infused sous vide steak	Yes	Kosher salt
Good Housekeeping	March 2021	761	Barley with sautéed leeks, peas & parsley	Yes	Kosher salt
Good Housekeeping	March 2021	762	Wheat berries with thyme-roasted grapes & spinach	Yes	Kosher salt
Good Housekeeping	March 2021	763	Parmesan egg-in-a-hole	Yes	Kosher salt
Good Housekeeping	March 2021	767	Quick chicken mole	Yes	Kosher salt
Good Housekeeping	March 2021	769	Chocolate hazelnut spread	Yes	Kosher salt
Good Housekeeping	March 2021	771	Jerk shrimp wraps with mango slaw	Yes	Kosher salt
Good Housekeeping	March 2021	772	Jerk shrimp wraps with mango slaw: jerk spice mix	Yes	Kosher salt
Good Housekeeping	March 2021	773	Coconut-crusted chicken cutlets	Yes	Kosher salt

Good Housekeeping	March 2021	774	Philly cheesesteaks	Yes	Kosher salt
Good Housekeeping	March 2021	776	Pulled pork nachos	Yes	Kosher salt
Good Housekeeping	March 2021	777	Greek chicken grain bowl	Yes	Kosher salt
Good Housekeeping	March 2021	780	Tex-Mex chicken salad	Yes	Kosher salt
Good Housekeeping	March 2021	781	Salmon Niçoise	Yes	Kosher salt
Good Housekeeping	March 2021	782	Cobb salad	Yes	Kosher salt
Good Housekeeping	March 2021	783	Sweet potato & black bean tacos	Yes	Kosher salt
Good Housekeeping	March 2021	784	White bean tuna salad	Yes	Kosher salt
Good Housekeeping	June 2021	786	Savory oatmeal with sautéed mushrooms and spinach	Yes	Kosher salt
Good Housekeeping	June 2021	789	Roasted strawberries	Yes	Kosher salt
Good Housekeeping	June 2021	790	Shrimp, zucchini and corn enchiladas	Yes	Kosher salt
Good Housekeeping	June 2021	792	Grilled butterflied chicken: for chicken	Yes	Kosher salt
Good Housekeeping	June 2021	793	Grilled butterflied chicken: for grilled scallions and citrus	Yes	Kosher salt
Good Housekeeping	June 2021	794	Crispy chickpeas	Yes	Kosher salt

Good Housekeepin g	June 2021	795	Caesar salad with grilled radishes: for vegan Caesar dressing	Yes	Kosher salt
Good Housekeepin g	June 2021	796	Caesar salad with grilled radishes: for salad	Yes	Kosher salt
Good Housekeepin g	June 2021	797	Gingery pork-meatball subs	Yes	Kosher salt
Good Housekeepin g	June 2021	798	Sheet pan chicken fajitas	Yes	Kosher salt
Good Housekeepin g	June 2021	800	Ricotta & fresh tomato pizza	Yes	Kosher salt
Good Housekeepin g	June 2021	801	Zucchini, yellow squash & herb pizza	Yes	Kosher salt
Good Housekeepin g	June 2021	804	Strawberries: pickled-berry toast	Yes	Kosher salt
Good Housekeepin g	June 2021	805	Spiced cod with rice noodle salad	Yes	Kosher salt
Good Housekeepin g	June 2021	806	Barley salad with strawberries and buttermilk dressing	Yes	Kosher salt
Good Housekeepin g	June 2021	807	Marinated flank steak	Yes	Kosher salt
Good Housekeepin g	June 2021	808	Grilled broccoli	Yes	Kosher salt
Good Housekeepin g	July/August 2021	813	White bean & broccolini salad	Yes	Kosher salt
Good Housekeepin g	July/August 2021	814	Marinated mushrooms	Yes	Kosher salt
Good Housekeepin g	July/August 2021	815	Heirloom tomato salad	Yes	Kosher salt

Good Housekeeping	July/August 2021	822	Summer succotash	Yes	Kosher salt
Good Housekeeping	July/August 2021	823	Barbecue ribs	Yes	Kosher salt
Good Housekeeping	July/August 2021	830	Pecan crumble	Yes	Kosher salt
Good Housekeeping	July/August 2021	831	Grilled fajita kebabs	Yes	Kosher salt
Good Housekeeping	July/August 2021	832	Pasta with no-cook tomato sauce	Yes	Kosher salt
Good Housekeeping	July/August 2021	833	Grilled peach caprese	Yes	Kosher salt
Good Housekeeping	January/February 2021	838	Spinach & artichoke baked pasta	Yes	Kosher salt
Good Housekeeping	January/February 2021	839	Cheesy jalapeño pretzel bites	Yes	Kosher salt
Good Housekeeping	January/February 2021	842	Chocolate heart cake	Yes	Kosher salt
Good Housekeeping	January/February 2021	845	Warm pizza dip	Yes	Kosher salt
Good Housekeeping	January/February 2021	846	Make-ahead egg-and-cheese sandwiches	Yes	Kosher salt
Good Housekeeping	January/February 2021	847	BBQ beef & mashed potato pie	Yes	Kosher salt
Good Housekeeping	January/February 2021	849	Preserved lemons	Yes	Kosher salt
Good Housekeeping	January/February 2021	852	Tunisian skillet chicken	Yes	Kosher salt

Good Housekeeping	December 2020	857	Dashing through the snow sugar cookies	Yes	Kosher salt
Good Housekeeping	December 2020	858	Chocolate chip cookie Santa sandwiches	Yes	Kosher salt
Good Housekeeping	December 2020	859	Cranberry-pear tart	Yes	Kosher salt
Good Housekeeping	December 2020	860	Vegetable torte	Yes	Kosher salt
Good Housekeeping	December 2020	861	Classic latkes	Yes	Kosher salt
Good Housekeeping	December 2020	867	Easy pork ragu	Yes	Kosher salt
Good Housekeeping	December 2020	868	Hot pepper & onion pizza	Yes	Kosher salt
Good Housekeeping	December 2020	872	Jammy ornament cutouts	Yes	Kosher salt
Good Housekeeping	December 2020	876	Apple- and apricot-stuffed pork loin	Yes	Kosher salt
Good Housekeeping	December 2020	877	Potato gnocchi with meat sauce	Yes	Kosher salt
Good Housekeeping	December 2020	878	Best-ever holiday eggnog	Yes	Kosher salt
Good Housekeeping	December 2020	881	Twice-baked citrus-almond brioche	Yes	Kosher salt
Good Housekeeping	December 2020	882	Brussels sprout salad with hazelnuts	Yes	Kosher salt
Good Housekeeping	December 2020	885	Gingerbread cookies for houses	Yes	Kosher salt

Good Housekeeping	April 2021	886	Carrot-pineapple cupcakes: for cupcakes	Yes	Kosher salt
Good Housekeeping	April 2021	889	Meyer lemon madeleines	Yes	Kosher salt
Good Housekeeping	April 2021	890	Silky coconut cream pie	Yes	Kosher salt
Good Housekeeping	April 2021	891	Flaky pie crust	Yes	Kosher salt
Good Housekeeping	April 2021	892	Slow-cooker porridge	Yes	Kosher salt
Good Housekeeping	April 2021	893	Pea fritters with shrimp salad	Yes	Kosher salt
Good Housekeeping	April 2021	894	Dill-crusted pork and farro salad	Yes	Kosher salt
Good Housekeeping	April 2021	895	Mashed potatoes and greens	Yes	Kosher salt
Good Housekeeping	April 2021	896	Ham & cheese bread pudding	Yes	Kosher salt
Good Housekeeping	April 2021	897	Easy Easter roast	Yes	Kosher salt
Good Housekeeping	April 2021	899	Mexican skirt steak and avocado salad	Yes	Kosher salt
Good Housekeeping	April 2021	900	Spring vegetable pizza	Yes	Kosher salt
Good Housekeeping	April 2021	902	Sugared lavender cupcakes: for cupcakes	Yes	Kosher salt
Good Housekeeping	April 2021	905	Pecan berry bursts	Yes	Kosher salt

Good Housekeeping	April 2021	906	Crispy mustard chicken with spring potato salad	Yes	Kosher salt
Good Housekeeping	April 2021	910	Greek yogurt pancakes	Yes	Kosher salt
Good Housekeeping	April 2021	911	Garlicky roasted radish bruschetta	Yes	Kosher salt
Good Housekeeping	April 2021	912	Dill dip	Yes	Kosher salt
Good Housekeeping	April 2021	913	Charred scallion tart	Yes	Kosher salt
Good Housekeeping	April 2021	914	Spring green panzanella	Yes	Kosher salt
Good Housekeeping	April 2021	915	Roasted asparagus with feta vinaigrette	Yes	Kosher salt
Good Housekeeping	July/August 2021		Blueberry crème fraîche semifreddo with pecan crumble: blueberry sauce/filling	Yes	Kosher salt
Good Housekeeping	July/August 2021		Blueberry crème fraîche semifreddo with pecan crumble: semifreddo base	Yes	Kosher salt
Good Housekeeping	July/August 2021		Green slaw: coleslaw dressing	Yes	Kosher salt
Good Housekeeping	December 2020		Roasted orange-ginger chicken and mushrooms	Yes	Kosher salt
Good Housekeeping	November 2020		Herb stuffing	Yes	Kosher salt
Good Housekeeping	October 2020		Caramel apple pie	Yes	Kosher salt
People	27 September 2021	628	Pizza with eggs, zucchini & salami	Yes	Kosher salt
People	27 September 2021	629	Key lime pie with coconut whipped cream	Yes	Kosher salt

People	20 September 2021	630	Guacamole with serrano & cayenne	Yes	Kosher salt
People	20 September 2021	631	Broccoli rabe pesto pasta	Yes	Kosher salt
People	13 September 2021	632	Horseradish-cheddar burgers	Yes	Kosher salt
People	13 September 2021	633	Chilled Greek watermelon salad	Yes	Kosher salt
People	11 October 2021	635	Pork & vegetable lo mein	Yes	Kosher salt
People	11 October 2021	636	French onion soup	Yes	Kosher salt
People	4 October 2021	638	Mapo pork with hummus	Yes	Kosher salt
People	4 October 2021	639	Spaghetti with lentils	Yes	Kosher salt
People	30 August 2021	644	Barbecue pork spareribs	Yes	Kosher salt
People	23 August 2021	646	Latin-Caribbean braised chicken with garlic rice	Yes	Kosher salt
People	16 August 2021	647	Crispy coconut & curry shrimp	Yes	Kosher salt
People	16 August 2021	648	Korean-inspired sloppy joes with quick pickles	Yes	Kosher salt
People	16 August 2021	649	Rosemary & garlic pork tenderloin	Yes	Kosher salt
People	2 August 2021	652	Grilled skirt steak nachos	Yes	Kosher salt
US Weekly	11 October 2021	1002	Stuffed pesto chicken breast	Yes	Kosher salt
US Weekly	28 June 2021		Beet and brown rice burgers	Yes	Kosher salt
Taste of Home	December/January 2021	255	Cream cheese red velvet thumbprint cookies	Yes	Kosher salt
Taste of Home	December/January 2021	260	The best hummus	Yes	Kosher salt
Taste of Home	February/March 2021	287	Easy one-dish king cake	Yes	Kosher salt
Taste of Home	February/March 2021	296	Guacamole	Yes	Kosher salt
Taste of Home	April/May 2021	369	Polpette di Mamma	Yes	Kosher salt
Taste of Home	June/July 2021	390	Watermelon pizza	Yes	Kosher salt
Taste of Home	June/July 2021	438	Farro salad with charred shishito peppers & corn	Yes	Kosher salt
Taste of Home	June/July 2021	441	Cherry tomato pasta with avocado sauce	Yes	Kosher salt
Taste of Home	August/September 2021	462	Beef short ribs vindaloo	Yes	Kosher salt

Taste of Home	August/September 2021	464	Grandma's apple cake	Yes	Kosher salt
Taste of Home	August/September 2021	489	Brown sugar bacon BLT sandwiches	Yes	Kosher salt
Taste of Home	October/November 2021	502	Pumpkin chocolate tart with cinnamon whipped cream	Yes	Kosher salt
Taste of Home	October/November 2021	504	Simple roast brussels sprouts	Yes	Kosher salt
Better Homes & Gardens	March 2021	4	Black & white cookies	Yes	Kosher salt
Better Homes & Gardens	March 2021	6	Bittersweet chocolate cake	Yes	Kosher salt
Better Homes & Gardens	March 2021	8	English lemon posset	Yes	Kosher salt
Better Homes & Gardens	April 2021	40	Tomate-caper sauce: Sicilian cod with tomato-caper sauce	Yes	Kosher salt
Better Homes & Gardens	May 2021	51	Hatch chile mac 'n' cheese	Yes	Kosher salt
Better Homes & Gardens	August 2021	91	Grandma-style pizza with capocollo & fennel: homemade pizza dough	Yes	Kosher salt
Better Homes & Gardens	September 2021	122	Jammy oat squares	Yes	Kosher salt
Better Homes & Gardens	September 2021	123	Chewy granola bites	Yes	Kosher salt
Better Homes & Gardens	October 2020	527	Oven beef & barley stew	Yes	Kosher salt
Better Homes & Gardens	November 2020	537	Twice-toasted farro & mushroom dressing	Yes	Kosher salt
Better Homes & Gardens	November 2020	554	Roasted squash	Yes	Kosher salt
Better Homes & Gardens	November 2020	555	Roasted squash: Macadamia nut gremolata	Yes	Kosher salt
Better Homes & Gardens	November 2020	558	Honeyed apricot-lime vinaigrette	Yes	Kosher salt
Better Homes & Gardens	November 2020	560	Master the mash: Celery root puree	Yes	Kosher salt
Better Homes & Gardens	November 2020	561	Master the mash: Mashed cauliflower	Yes	Kosher salt

Better Homes & Gardens	December 2020	565	Jerk chicken	Yes	Kosher salt
Better Homes & Gardens	February 2021		Pesto pasta with charred radicchio: any herb pesto	Yes	Kosher salt
Better Homes & Gardens	February 2021		Roasted garlic-goat cheese pizza: basil vinaigrette	Yes	Kosher salt
Good Housekeeping	July/August 2021	820	Herby potato salad	Yes	Kosher salt and flaky sea salt
Good Housekeeping	July/August 2021	821	Watermelon salad	Yes	Kosher salt and flaky sea salt
Good Housekeeping	December 2020	884	Celebration chocolate-walnut cookies	Yes	Kosher salt and flaky sea salt
AARP	December 2019/January 2020	941	Meat loaf remix: Texas BBQ	No	Not applicable
AARP	December 2019/January 2020	942	Meat loaf remix: Wisconsin cheddar	No	Not applicable
AARP	December 2019/January 2020	943	Meat loaf remix: California vegan	No	Not applicable
AARP	December 2019/January 2020	944	Meat loaf remix: Korean spicy	No	Not applicable
AARP	December 2019/January 2020	945	Meat loaf remix: South African bobotie	No	Not applicable
AARP	December 2019/January 2020	946	Meat loaf remix: Greek lamb	No	Not applicable
AARP	October/November 2020	931	The ultimate immunity smoothie	No	Not applicable
AARP	April/May 2021	919	12 new uses for strawberries: rice pilaf	No	Not applicable
AARP	April/May 2021	920	12 new uses for strawberries: pancakes	No	Not applicable
AARP	April/May 2021	921	12 new uses for strawberries: ice cubes	No	Not applicable
AARP	April/May 2021	922	12 new uses for strawberries: tasty spread	No	Not applicable
AARP	April/May 2021	923	12 new uses for strawberries: frozen yogurt	No	Not applicable
AARP	April/May 2021	924	12 new uses for strawberries: salsa	No	Not applicable

AARP	April/May 2021	925	12 new uses for strawberries: lemonade	No	Not applicable
AARP	April/May 2021	927	12 new uses for strawberries: pastry	No	Not applicable
AARP	April/May 2021	928	12 new uses for strawberries: salad	No	Not applicable
AARP	April/May 2021	930	12 new uses for strawberries: dessert bites	No	Not applicable
AARP	June/July 2021	933	Grill and chill: grilled tofu	No	Not applicable
AARP	June/July 2021	934	Grill and chill: drunken chicken	No	Not applicable
AARP	June/July 2021	935	Grill and chill: a mountain of burgers	No	Not applicable
AARP	June/July 2021	936	Grill and chill: pork shoulder	No	Not applicable
AARP	August/September 2021	947	6 delicious uses for a muffin tin: frittatas	No	Not applicable
AARP	August/September 2021	948	6 delicious uses for a muffin tin: fruit pies	No	Not applicable
AARP	August/September 2021	949	6 delicious uses for a muffin tin: potato gratin	No	Not applicable
AARP	August/September 2021	950	6 delicious uses for a muffin tin: taco-salad cups	No	Not applicable
AARP	August/September 2021	951	6 delicious uses for a muffin tin: mini pizzas	No	Not applicable
AARP	August/September 2021	952	6 delicious uses for a muffin tin: mini meat loaves	No	Not applicable
Cosmopolitan	May/June 2021	955	Choose your own egg adventure: egg drop soup	No	Not applicable
Cosmopolitan	May/June 2021	956	Choose your own egg adventure: lowkey shakshuka	No	Not applicable
Cosmopolitan	May/June 2021	958	Choose your own egg adventure: a frittata!	No	Not applicable
Cosmopolitan	July/August 2021	960	This DIY vegan ice cream tastes like a million bucks	No	Not applicable
Cosmopolitan	December 2020/January 2021	962	These v unclassy desserts make total sense right now: peppermint bark hot chocolate	No	Not applicable

Cosmopolitan	December 2020/January 2021	963	These v unclassy desserts make total sense right now: peppermint-and-candy-cane-dipped oreos	No	Not applicable
Cosmopolitan	December 2020/January 2021	964	These v unclassy desserts make total sense right now: mischief munch	No	Not applicable
Cosmopolitan	September 2021	965	Homemade dipped cones are your love language now	No	Not applicable
Cosmopolitan	September 2021	967	The fluffiest pancakes of your freakin' life	No	Not applicable
Cosmopolitan	October 2021	971	Watch me turn frozen chicken nuggets into fancy apps that'll impress your friends: teeny chicken and waffles	No	Not applicable
Cosmopolitan	October 2021	972	Watch me turn frozen chicken nuggets into fancy apps that'll impress your friends: BB chicken sammies	No	Not applicable
Cosmopolitan	October 2021	973	Watch me turn frozen chicken nuggets into fancy apps that'll impress your friends: lil chicken parm bites	No	Not applicable
Cosmopolitan	October 2020	976	If you're not putting Halloween candy in your brownies, WTD?!	No	Not applicable
Cosmopolitan	November 2020	982	Breakfast tacos: the laziest healthy meal that doesn't tase like dirt: got avocados?	No	Not applicable
Cosmopolitan	November 2020	983	Breakfast tacos: the laziest healthy meal that doesn't tase like dirt: got beans	No	Not applicable
Cosmopolitan	November 2020	984	Breakfast tacos: the laziest healthy meal that doesn't tase like dirt: got frozen tots?	No	Not applicable

Cosmopolitan	March 2021	986	Just a list of ways to go from *not* having a stupidly good St. Pat's Day shake to *having* a stupidly good St. Pat's Day shake	No	Not applicable
Cosmopolitan	June 2020	989	DIY dipping sauces that make veggies taste like takeout: vegan buffalo sauce	No	Not applicable
Cosmopolitan	April 2021		No recipes in issue	Not applicable	Not applicable
Cosmopolitan	July/August 2020		No recipes in issue	Not applicable	Not applicable
Good Housekeeping	September 2021	654	Spicy shrimp lettuce wraps	No	Not applicable
Good Housekeeping	September 2021	660	Pea and leek pasta	No	Not applicable
Good Housekeeping	September 2021	661	Lemony couscous	No	Not applicable
Good Housekeeping	September 2021	662	Stewy beans & shrimp	No	Not applicable
Good Housekeeping	September 2021	663	Garlicky beans	No	Not applicable
Good Housekeeping	September 2021	670	Crispy pork noodles	No	Not applicable
Good Housekeeping	October 2020	676	"Bloody" bites cake pops	No	Not applicable
Good Housekeeping	October 2020	678	Chocolate ghostly tarts: for filling	No	Not applicable
Good Housekeeping	October 2020	679	Chocolate ghostly tarts: for ghosts	No	Not applicable
Good Housekeeping	October 2020	681	Cider sangria	No	Not applicable

Good Housekeeping	October 2020	683	Caramel apple pie: for apples	No	Not applicable
Good Housekeeping	October 2020	689	Seafood, chorizo & vegetable stew	No	Not applicable
Good Housekeeping	November 2020	715	Fig & rosemary cranberry sauce	No	Not applicable
Good Housekeeping	November 2020	717	Orange-buttermilk chess pie: candied orange slices	No	Not applicable
Good Housekeeping	November 2020	718	Cherry-berry jumble fruit pie	No	Not applicable
Good Housekeeping	November 2020	723	Decadent chocolate-espresso pie: for whipped cream	No	Not applicable
Good Housekeeping	November 2020	725	Orecchiette with white beans and spinach	No	Not applicable
Good Housekeeping	November 2020	728	Tangy cranberry meatballs	No	Not applicable
Good Housekeeping	May 2021	735	Beef and broccoli	No	Not applicable
Good Housekeeping	May 2021	739	Lemon bundt cake: for icing	No	Not applicable
Good Housekeeping	May 2021	741	Thin mint cheesecake: for cheesecake	No	Not applicable
Good Housekeeping	May 2021	745	Mediterranean olive sauce	No	Not applicable
Good Housekeeping	May 2021	749	Buffalo wing burger: for spicy mayo	No	Not applicable
Good Housekeeping	May 2021	755	Cauliflower fried rice	No	Not applicable

Good Housekeeping	May 2021	757	Artichokes: to steam	No	Not applicable
Good Housekeeping	March 2021	760	Lemony chicken & rice soup	No	Not applicable
Good Housekeeping	March 2021	764	Mint: lemon-mint slushie	No	Not applicable
Good Housekeeping	March 2021	768	Chocolate hazelnut cheesecake	No	Not applicable
Good Housekeeping	March 2021	770	Pecan sticky buns	No	Not applicable
Good Housekeeping	March 2021	775	Vegetarian fried rice	No	Not applicable
Good Housekeeping	March 2021	778	Spice-grilled steak salad	No	Not applicable
Good Housekeeping	March 2021	779	Shrimp soba salad	No	Not applicable
Good Housekeeping	June 2021	785	Raspberry-chia jam	No	Not applicable
Good Housekeeping	June 2021	787	Best ever French toast	No	Not applicable
Good Housekeeping	June 2021	788	Roasted strawberries and brie	No	Not applicable
Good Housekeeping	June 2021	791	Ice cream float	No	Not applicable
Good Housekeeping	June 2021	799	Lemon cacio e pepe pizza	No	Not applicable
Good Housekeeping	June 2021	802	Strawberries: sparkling lemonade	No	Not applicable

Good Housekeeping	June 2021	803	Strawberries: cheesecake ice cream	No	Not applicable
Good Housekeeping	June 2021	809	Orange-spiked chimichurri	No	Not applicable
Good Housekeeping	June 2021	810	Marinade trio: spicy soy	No	Not applicable
Good Housekeeping	June 2021	811	Marinade trio: citrus garlic	No	Not applicable
Good Housekeeping	June 2021	812	Marinade trio: balsamic rosemary	No	Not applicable
Good Housekeeping	July/August 2021	816	Agua fresca	No	Not applicable
Good Housekeeping	July/August 2021	817	Pineapple-cucumber smoothie	No	Not applicable
Good Housekeeping	July/August 2021	818	Peach-mango smoothie	No	Not applicable
Good Housekeeping	July/August 2021	819	Green slaw	No	Not applicable
Good Housekeeping	July/August 2021	824	Corn: quick chowder	No	Not applicable
Good Housekeeping	July/August 2021	827	Cherry cocktail syrup	No	Not applicable
Good Housekeeping	January/February 2021		Chocolate heart cake: frosting and decorations	No	Not applicable
Good Housekeeping	January/February 2021	853	Easy maple horchata	No	Not applicable
Good Housekeeping	December 2020	869	Coco razz smoothie	No	Not applicable

Good Housekeeping	December 2020	873	Eggnog truffles	No	Not applicable
Good Housekeeping	December 2020	874	Ham & spinach pizza	No	Not applicable
Good Housekeeping	December 2020	880	Mary Pickford	No	Not applicable
Good Housekeeping	December 2020	883	Cranberry swirl cheesecake bars	No	Not applicable
Good Housekeeping	April 2021	887	Carrot-pineapple cupcakes: for frosting	No	Not applicable
Good Housekeeping	April 2021	888	Pineapple flowers	No	Not applicable
Good Housekeeping	April 2021	898	Elderflower gin spritz	No	Not applicable
Good Housekeeping	April 2021	901	Easter cookies & cream mini cheesecakes	No	Not applicable
Good Housekeeping	April 2021	903	Sugared lavender cupcakes: for glaze	No	Not applicable
Good Housekeeping	April 2021	904	Sugared lavender cupcakes: sugared lavender	No	Not applicable
Good Housekeeping	April 2021	909	Scallions: creamy rice	No	Not applicable
Good Housekeeping	December 2020		Butternut squash + mushroom enchiladas	No	Not applicable
Good Housekeeping	October 2020		Chocolate ghostly tarts: for shells	No	Not applicable
Good Housekeeping	October 2020		Witches' brew	No	Not applicable

Good Housekeeping	July/August 2020		Sweet + tart side	No	Not applicable
Good Housekeeping	July/August 2020		Smoky dessert	No	Not applicable
People	6 September 2021	634	Papaya salad with shrimp	No	Not applicable
People	23 August 2021	645	Coconut & berry skillet French toast	No	Not applicable
People	9 August 2021	650	Beef bulgogi lettuce wraps	No	Not applicable
US Weekly	30 August 2021	994	Cauliflower buffalo wings	No	Not applicable
US Weekly	2 August 2021	1005	Busy's bloody Mary	No	Not applicable
US Weekly	2 August 2021	1006	Italian getaway	No	Not applicable
US Weekly	1 November 2021		No recipes in issue	Not applicable	Not applicable
US Weekly	4 October 2021		No recipes in issue	Not applicable	Not applicable
US Weekly	27 September 2021		No recipes in issue	Not applicable	Not applicable
US Weekly	20 September 2021		No recipes in issue	Not applicable	Not applicable
US Weekly	13 September 2021		No recipes in issue	Not applicable	Not applicable
US Weekly	6 September 2021		No recipes in issue	Not applicable	Not applicable
US Weekly	16 August 2021		No recipes in issue	Not applicable	Not applicable
US Weekly	26 July 2021		No recipes in issue	Not applicable	Not applicable
US Weekly	19 July 2021		No recipes in issue	Not applicable	Not applicable
US Weekly	12 July 2021		No recipes in issue	Not applicable	Not applicable
US Weekly	21 June 2021		No recipes in issue	Not applicable	Not applicable
US Weekly	14 June 2021		No recipes in issue	Not applicable	Not applicable
US Weekly	7 June 2021		No recipes in issue	Not applicable	Not applicable
US Weekly	9 August 2021	1003	Ham & cheese quesadilla	No	Not applicable

US Weekly	23 August 2021		Funfetti flan cake	No	Not applicable
US Weekly	5 July 2021		No recipes in issue	Not applicable	Not applicable
Vanity Fair	November 2021		No recipes in issue	Not applicable	Not applicable
Vanity Fair	October 2021		No recipes in issue	Not applicable	Not applicable
Vanity Fair	September 2021		No recipes in issue	Not applicable	Not applicable
Vanity Fair	July/August 2021		No recipes in issue	Not applicable	Not applicable
Vanity Fair	June 2021		No recipes in issue	Not applicable	Not applicable
Vanity Fair	May 2021		No recipes in issue	Not applicable	Not applicable
Vanity Fair	April 2021		No recipes in issue	Not applicable	Not applicable
Vanity Fair	Hollywood 2021		No recipes in issue	Not applicable	Not applicable
Vanity Fair	March 2021		No recipes in issue	Not applicable	Not applicable
Vanity Fair	Holiday 2020/2021		No recipes in issue	Not applicable	Not applicable
Vanity Fair	December 2020		No recipes in issue	Not applicable	Not applicable
Vanity Fair	October 2020		No recipes in issue	Not applicable	Not applicable
Vanity Fair	November 2020		No recipes in issue	Not applicable	Not applicable
Taste of Home	August/September 2020	126	Bacon-chicken club pizza	No	Not applicable
Taste of Home	August/September 2020	128	Swiss chicken sliders	No	Not applicable
Taste of Home	August/September 2020	130	Slow-cooker meatball sandwiches	No	Not applicable
Taste of Home	August/September 2020	131	Tangy sweet-and-sour meatballs	No	Not applicable
Taste of Home	August/September 2020	134	Weeknight goulash	No	Not applicable
Taste of Home	August/September 2020	137	Flourless chocolate torte	No	Not applicable
Taste of Home	August/September 2020	138	Easy white chicken chili	No	Not applicable

Taste of Home	August/September 2020	139	Pineapple shrimp fried rice	No	Not applicable
Taste of Home	August/September 2020	141	Apricot-ginger acorn squash	No	Not applicable
Taste of Home	August/September 2020	147	Cinnamon apple cider monkey bread	No	Not applicable
Taste of Home	August/September 2020	154	Nacho pie	No	Not applicable
Taste of Home	August/September 2020	159	Habanero strawberry jam	No	Not applicable
Taste of Home	August/September 2020	161	One-pan sweet chili shrimp & veggies	No	Not applicable
Taste of Home	August/September 2020	163	One-pot red beans & rice	No	Not applicable
Taste of Home	August/September 2020	165	Easy chicken pesto stuffed peppers	No	Not applicable
Taste of Home	August/September 2020	167	Fruit smoothie bowls	No	Not applicable
Taste of Home	August/September 2020	168	Coconut cold brew latte	No	Not applicable
Taste of Home	August/September 2020	169	Cheesy roast beef pinwheels	No	Not applicable
Taste of Home	October/November 2020	171	Angel hair pasta with spinach & spinach	No	Not applicable
Taste of Home	October/November 2020	172	Chicken biscuit skillet	No	Not applicable
Taste of Home	October/November 2020	173	Indian-spiced beefy lettuce wraps	No	Not applicable
Taste of Home	October/November 2020	179	Quick tomato soup	No	Not applicable
Taste of Home	October/November 2020	181	Sugared cranberries	No	Not applicable
Taste of Home	October/November 2020	184	Traditional holiday stuffing	No	Not applicable
Taste of Home	October/November 2020	185	Corn pudding	No	Not applicable
Taste of Home	October/November 2020	186	Hot spiced cranberry drink	No	Not applicable
Taste of Home	October/November 2020	191	Mashed cauliflower with parmesan	No	Not applicable
Taste of Home	October/November 2020	198	Ready, set...roll!: buffalo	No	Not applicable

Taste of Home	October/November 2020	199	Ready, set...roll!: nacho	No	Not applicable
Taste of Home	October/November 2020	200	Ready, set...roll!: pesto	No	Not applicable
Taste of Home	October/November 2020	201	Ready, set...roll!: reuben	No	Not applicable
Taste of Home	October/November 2020	202	Ready, set...roll!: feta & oliva	No	Not applicable
Taste of Home	October/November 2020	203	Ready,set...roll!: curried-apricot	No	Not applicable
Taste of Home	October/November 2020	204	Ready, set...roll!: smoked salmon	No	Not applicable
Taste of Home	October/November 2020	205	Ready, set...roll!: chicken noodle	No	Not applicable
Taste of Home	October/November 2020	206	Ready, set...roll!: pepperoni pizza	No	Not applicable
Taste of Home	October/November 2020	207	Ready, set...roll!: blue cheese	No	Not applicable
Taste of Home	October/November 2020	208	The best quiche Lorraine	No	Not applicable
Taste of Home	October/November 2020	217	Lake Charles dip	No	Not applicable
Taste of Home	October/November 2020	218	Cranberry cherry punch	No	Not applicable
Taste of Home	October/November 2020	220	Chocolate caramel cookies	No	Not applicable
Taste of Home	October/November 2020	222	Fried banana milkshakes	No	Not applicable
Taste of Home	December/January 2021	223	Warm grapefruit with ginger sugar	No	Not applicable
Taste of Home	December/January 2021	224	Madras curried eggs with rice	No	Not applicable
Taste of Home	December/January 2021	225	Quick & easy chicken poke bowl	No	Not applicable
Taste of Home	December/January 2021	227	Sausage & pancake bake	No	Not applicable
Taste of Home	December/January 2021	231	Haute chocolate: biscoff	No	Not applicable
Taste of Home	December/January 2021	232	Haute chocolate: chai	No	Not applicable
Taste of Home	December/January 2021	233	Haute chocolate: chili-orange	No	Not applicable

Taste of Home	December/January 2021	234	Haute chocolate: creamy white	No	Not applicable
Taste of Home	December/January 2021	235	Haute chocolate: hazelnut mocha	No	Not applicable
Taste of Home	December/January 2021	236	Haute chocolate: heavenly nutmeg	No	Not applicable
Taste of Home	December/January 2021	237	Haute chocolate: peppermint red velvet	No	Not applicable
Taste of Home	December/January 2021	238	Haute chocolate: pumpkin spice	No	Not applicable
Taste of Home	December/January 2021	239	Haute chocolate: salted caramel & banana	No	Not applicable
Taste of Home	December/January 2021	240	Haute chocolate: snickerdoodle	No	Not applicable
Taste of Home	December/January 2021	242	Crispy Christmas trees	No	Not applicable
Taste of Home	December/January 2021	243	Goody-goody gumdrops	No	Not applicable
Taste of Home	December/January 2021	244	Mulled wine margaritas	No	Not applicable
Taste of Home	December/January 2021	245	Eggnog tres leches cake	No	Not applicable
Taste of Home	December/January 2021	246	Caramel heavenlies	No	Not applicable
Taste of Home	December/January 2021	248	Peppermint cheesecake on a stick	No	Not applicable
Taste of Home	December/January 2021	252	Brie cherry pastry cups	No	Not applicable
Taste of Home	December/January 2021	253	Spiral ham with cranberry glaze	No	Not applicable
Taste of Home	December/January 2021	256	Peanut butter cinnamon snap cookies	No	Not applicable
Taste of Home	December/January 2021	259	Rolled butter almond cookies	No	Not applicable
Taste of Home	December/January 2021	262	Two-tone caramel brownies	No	Not applicable
Taste of Home	December/January 2021	264	Pumpkin French toast with bacon maple syrup	No	Not applicable
Taste of Home	December/January 2021	266	Holiday pretzel salad	No	Not applicable
Taste of Home	December/January 2021	267	Hot shrimp dip	No	Not applicable

Taste of Home	December/January 2021	270	Minty cookies & cream chocolate fudge	No	Not applicable
Taste of Home	December/January 2021	273	Hawaiian egg rolls	No	Not applicable
Taste of Home	December/January 2021	274	Easy coconut shrimp	No	Not applicable
Taste of Home	December/January 2021	275	Nutty Hawaiian	No	Not applicable
Taste of Home	December/January 2021	276	Peppermint popcorn	No	Not applicable
Taste of Home	February/March 2021	279	Banana fudge pie	No	Not applicable
Taste of Home	February/March 2021	281	Ramen noodle stir-fry	No	Not applicable
Taste of Home	February/March 2021	283	Tex-Mex grain bowl	No	Not applicable
Taste of Home	February/March 2021	285	Poppy seed chicken	No	Not applicable
Taste of Home	February/March 2021	290	Salted peanut squares	No	Not applicable
Taste of Home	February/March 2021	291	Triple berry mini pies	No	Not applicable
Taste of Home	February/March 2021	292	Red velvet cake in a jar	No	Not applicable
Taste of Home	February/March 2021	294	Rose water rice pudding	No	Not applicable
Taste of Home	February/March 2021	295	Lebanese stuffed cabbages	No	Not applicable
Taste of Home	February/March 2021	297	Guacamole: radish + mandarin orange	No	Not applicable
Taste of Home	February/March 2021	298	Guacamole: grilled chicken + cherry tomato	No	Not applicable
Taste of Home	February/March 2021	299	Guacamole: basil + toasted pine nuts	No	Not applicable
Taste of Home	February/March 2021	300	Guacamole: cajun shrimp + red pepper	No	Not applicable
Taste of Home	February/March 2021	301	Guacamole: mango + habanero	No	Not applicable
Taste of Home	February/March 2021	302	Guacamole: black bean + corn	No	Not applicable
Taste of Home	February/March 2021	303	Guacamole: blue cheese + toasted almonds	No	Not applicable

Taste of Home	February/March 2021	304	Guacamole: jicama + pineapple	No	Not applicable
Taste of Home	February/March 2021	305	Guacamole: bacon + cotija cheese	No	Not applicable
Taste of Home	February/March 2021	306	Guacamole: apple + white onion	No	Not applicable
Taste of Home	February/March 2021	307	Pepper jelly hogs in a blanket	No	Not applicable
Taste of Home	February/March 2021	309	Peanut butter & jelly cheesecake	No	Not applicable
Taste of Home	February/March 2021	313	Waffle-iron pizzas	No	Not applicable
Taste of Home	February/March 2021	317	Parisian sipping chocolate	No	Not applicable
Taste of Home	February/March 2021	323	Slow-cooker baked potatoes	No	Not applicable
Taste of Home	February/March 2021	325	Hot chili cheese dip	No	Not applicable
Taste of Home	February/March 2021	327	Chive butter balls	No	Not applicable
Taste of Home	April/May 2021	331	Lemon, ginger & turmeric infused water	No	Not applicable
Taste of Home	April/May 2021	332	Crumb-topped sole	No	Not applicable
Taste of Home	April/May 2021	333	Salmon croquette breakfast sandwich	No	Not applicable
Taste of Home	April/May 2021	337	Alfredo sauce: Fettucine alfredo	No	Not applicable
Taste of Home	April/May 2021	338	Alfredo sauce: Seafood alfredo	No	Not applicable
Taste of Home	April/May 2021	342	Homemade marinara sauce: Spicy bratwurst supper	No	Not applicable
Taste of Home	April/May 2021	344	Classic pesto: Easy pesto pizza	No	Not applicable
Taste of Home	April/May 2021	353	Sweet tea concentrate	No	Not applicable
Taste of Home	April/May 2021	354	Quick ambrosia fruit salad	No	Not applicable
Taste of Home	April/May 2021	355	Butter pecan cheesecake	No	Not applicable
Taste of Home	April/May 2021	356	Sunny citrus layered cheesecake	No	Not applicable

Taste of Home	April/May 2021	357	Double chocolate espresso cheesecake	No	Not applicable
Taste of Home	April/May 2021	358	New York cheescake with shortbread crust	No	Not applicable
Taste of Home	April/May 2021	359	Classic margarita	No	Not applicable
Taste of Home	April/May 2021	360	Melon margarita	No	Not applicable
Taste of Home	April/May 2021	361	Caribbean margarita	No	Not applicable
Taste of Home	April/May 2021	362	Amaretto margarita	No	Not applicable
Taste of Home	April/May 2021	363	Grapefruit sunset margarita	No	Not applicable
Taste of Home	April/May 2021	364	Blueberry-mint frozen margarita	No	Not applicable
Taste of Home	April/May 2021	365	Raspberry-ginger frozen margarita	No	Not applicable
Taste of Home	April/May 2021	366	Frozen coconut margarita	No	Not applicable
Taste of Home	April/May 2021	367	Strawberry-basil frozen margarita	No	Not applicable
Taste of Home	April/May 2021	368	Sriracha-mango frozen margarita	No	Not applicable
Taste of Home	April/May 2021	371	Candy bar fudge	No	Not applicable
Taste of Home	April/May 2021	373	Sheet-pan jambalaya with cauliflower rice	No	Not applicable
Taste of Home	April/May 2021	374	Breakfast BLT waffles	No	Not applicable
Taste of Home	April/May 2021	376	Strawberry overnight oats	No	Not applicable
Taste of Home	April/May 2021	378	Mean green smoothie bowls	No	Not applicable
Taste of Home	April/May 2021	382	Pear quinoa breakfast bake	No	Not applicable
Taste of Home	April/May 2021	383	Yoda soda	No	Not applicable
Taste of Home	April/May 2021	384	Robot cake	No	Not applicable
Taste of Home	April/May 2021	386	Lightsaber pretzels	No	Not applicable

Taste of Home	April/May 2021	387	Passion fruit mojito	No	Not applicable
Taste of Home	June/July 2021	389	Five-minute blueberry pie	No	Not applicable
Taste of Home	June/July 2021	392	Chicken strawberry spinach salad	No	Not applicable
Taste of Home	June/July 2021	394	Dad's lemony grilled chicken: Cheesy chicken quesadilla	No	Not applicable
Taste of Home	June/July 2021	395	Dad's lemony grilled chicken: Chicken fried rice	No	Not applicable
Taste of Home	June/July 2021	396	Dad's lemony grilled chicken: Buffalo chicken baked potato	No	Not applicable
Taste of Home	June/July 2021	397	Easy marinated grilled flank steak	No	Not applicable
Taste of Home	June/July 2021	398	Easy marinated grilled flank steak: Grilled flank steak salad	No	Not applicable
Taste of Home	June/July 2021	399	Easy marinated grilled flank steak: Philly cheesesteak sandwiches	No	Not applicable
Taste of Home	June/July 2021	400	Easy marinated grilled flank steak: Grilled steak tacos	No	Not applicable
Taste of Home	June/July 2021	402	Skewered lamb with blackberry-balsamic glaze: Greek mac & cheese	No	Not applicable
Taste of Home	June/July 2021	403	Skewered lamb with blackberry-balsamic glaze: Mediterranean grain bowl	No	Not applicable
Taste of Home	June/July 2021	404	Skewered lamb with blackberry-balsamic glaze: Lemony lamb fettucine	No	Not applicable
Taste of Home	June/July 2021	414	Tajin limeade	No	Not applicable
Taste of Home	June/July 2021	416	Hearty chicken enchiladas	No	Not applicable
Taste of Home	June/July 2021	418	Peanut butter silk pie	No	Not applicable

Taste of Home	June/July 2021	419	Berry pretzel fluff dessert	No	Not applicable
Taste of Home	June/July 2021	420	Peach Bavarian	No	Not applicable
Taste of Home	June/July 2021	421	Rainbow sherbet angel food cake	No	Not applicable
Taste of Home	June/July 2021	422	Grecian macaroni salad	No	Not applicable
Taste of Home	June/July 2021	423	Scandanavian macaroni salad	No	Not applicable
Taste of Home	June/July 2021	424	Caprese macaroni salad	No	Not applicable
Taste of Home	June/July 2021	425	Middle Eastern macaroni salad	No	Not applicable
Taste of Home	June/July 2021	426	Chicken caesar macaroni salad	No	Not applicable
Taste of Home	June/July 2021	427	Polynesian macaroni salad	No	Not applicable
Taste of Home	June/July 2021	428	Cubano macaroni salad	No	Not applicable
Taste of Home	June/July 2021	429	Chicken taco macaroni salad	No	Not applicable
Taste of Home	June/July 2021	430	Barbecue macaroni salad	No	Not applicable
Taste of Home	June/July 2021	431	Shrimp & crab macaroni salad	No	Not applicable
Taste of Home	June/July 2021	440	Roasted grape & sweet cheese phyllo galette	No	Not applicable
Taste of Home	June/July 2021	442	Citrus cantaloupe butter	No	Not applicable
Taste of Home	June/July 2021	443	Peaches & cream whiskey loaf	No	Not applicable
Taste of Home	June/July 2021	444	Grilled elote flatbread	No	Not applicable
Taste of Home	June/July 2021	448	Spicy Thai noodle watermelon salad	No	Not applicable
Taste of Home	June/July 2021	449	Creamy corn custards	No	Not applicable
Taste of Home	June/July 2021	451	Cheese & pimiento spread	No	Not applicable
Taste of Home	June/July 2021	452	Favorite hot chocolate	No	Not applicable

Taste of Home	June/July 2021	454	Lemon-apricot fruit pops	No	Not applicable
Taste of Home	August/September 2021	456	That's a wrap!	No	Not applicable
Taste of Home	August/September 2021	457	Blackberry shrub	No	Not applicable
Taste of Home	August/September 2021	458	Tarragon tuna salad	No	Not applicable
Taste of Home	August/September 2021	460	Shrimp tostadas with avocado salsa	No	Not applicable
Taste of Home	August/September 2021	469	Spaghetti with bacon	No	Not applicable
Taste of Home	August/September 2021	470	Crunchy burger quesadillas	No	Not applicable
Taste of Home	August/September 2021	472	Bacon breakfast cookies	No	Not applicable
Taste of Home	August/September 2021	479	Stir-fry scallops	No	Not applicable
Taste of Home	August/September 2021	481	Grilled pimiento cheese sandwiches	No	Not applicable
Taste of Home	August/September 2021	483	Hoisin chicken wraps	No	Not applicable
Taste of Home	August/September 2021	484	Kilbourn sandwich	No	Not applicable
Taste of Home	August/September 2021	487	Hot Italian party sandwiches	No	Not applicable
Taste of Home	August/September 2021	490	Currywurst	No	Not applicable
Taste of Home	October/November 2021	494	Piecaken	No	Not applicable
Taste of Home	October/November 2021	496	Easy ground beef stroganoff	No	Not applicable
Taste of Home	October/November 2021	498	Quick & easy skillet lasagna	No	Not applicable
Taste of Home	October/November 2021	500	Breakfast wraps	No	Not applicable
Taste of Home	October/November 2021	503	Maple-honey cranberry sauce	No	Not applicable
Taste of Home	October/November 2021	508	Cranberry apple slab pie	No	Not applicable
Taste of Home	October/November 2021	511	Sweet & puckery cupcakes	No	Not applicable

Taste of Home	October/November 2021	512	Peanut-cashew marshmallow pie	No	Not applicable
Taste of Home	October/November 2021	513	Make-ahead turkey gravy	No	Not applicable
Taste of Home	October/November 2021	514	Maple miso sweet potato casserole	No	Not applicable
Taste of Home	October/November 2021	519	Cherry lemon-lime punch	No	Not applicable
Taste of Home	October/November 2021	521	Confetti snack mix	No	Not applicable
Taste of Home	October/November 2021	522	Turkey, gouda & apple tea sandwiches	No	Not applicable
Better Homes & Gardens	March 2021	1	Matzo brei with lox and chives	No	Not applicable
Better Homes & Gardens	March 2021	2	Banana rum trifle	No	Not applicable
Better Homes & Gardens	March 2021	3	Banana rum trifle: sweetened whipped cream	No	Not applicable
Better Homes & Gardens	March 2021	5	Boston cream pie	No	Not applicable
Better Homes & Gardens	March 2021	9	Pot o' gold Guinness chocolate cupcakes	No	Not applicable
Better Homes & Gardens	March 2021	10	Italian chicken meatballs	No	Not applicable
Better Homes & Gardens	March 2021	11	Turkey pot pies	No	Not applicable
Better Homes & Gardens	March 2021	12	Macaroni and cheese with cashew cream	No	Not applicable
Better Homes & Gardens	March 2021	13	Macaroni and cheese with cashew cream: toasted bread crumbs	No	Not applicable
Better Homes & Gardens	March 2021	14	Open-face cheesesteaks	No	Not applicable
Better Homes & Gardens	March 2021	15	Sou bourek	No	Not applicable
Better Homes & Gardens	March 2021	16	Suugo suqaar	No	Not applicable
Better Homes & Gardens	March 2021	17	Suugo suqaar: xawaash spice mix	No	Not applicable
Better Homes & Gardens	March 2021	18	Pure rhubarb crumble pie	No	Not applicable

Better Homes & Gardens	March 2021	19	Pure rhubarb crumble pie: strawberry-rhubarb pie	No	Not applicable
Better Homes & Gardens	March 2021	20	All-buttah pie dough	No	Not applicable
Better Homes & Gardens	March 2021	21	Zarela's pineapple-ginger wings	No	Not applicable
Better Homes & Gardens	April 2021	22	Stuffed French toast: nut butter and bananas	No	Not applicable
Better Homes & Gardens	April 2021	23	Stuffed French toast: sausage and asparagus	No	Not applicable
Better Homes & Gardens	April 2021	24	Steamed artichokes	No	Not applicable
Better Homes & Gardens	April 2021	25	Fresh mint, basil, and pistachio sauce	No	Not applicable
Better Homes & Gardens	April 2021	26	Tarragon butter	No	Not applicable
Better Homes & Gardens	April 2021	27	Dijon dipping sauce	No	Not applicable
Better Homes & Gardens	April 2021	28	Raw artichoke salad with manchego cheese	No	Not applicable
Better Homes & Gardens	April 2021	29	Herb-and-pancetta-stuffed artichokes	No	Not applicable
Better Homes & Gardens	April 2021	30	Grilled baby artichoke antipasto	No	Not applicable
Better Homes & Gardens	April 2021	31	Orange bunny rolls	No	Not applicable
Better Homes & Gardens	April 2021	32	Lime crunch cheesecake	No	Not applicable
Better Homes & Gardens	April 2021	33	Carrot cake cream pies	No	Not applicable
Better Homes & Gardens	April 2021	34	Malted brownies	No	Not applicable
Better Homes & Gardens	April 2021	35	Malted brownies: malted milk frosting	No	Not applicable
Better Homes & Gardens	April 2021	36	Verde simmer sauce	No	Not applicable
Better Homes & Gardens	April 2021	37	Verde simmer sauce: black bean skillet enchiladas verde	No	Not applicable
Better Homes & Gardens	April 2021	38	Verde simmer sauce: chicken verde tacos	No	Not applicable

Better Homes & Gardens	April 2021	39	Tomato-caper sauce	No	Not applicable
Better Homes & Gardens	April 2021	41	Tomate-caper sauce: tortellini & smoked sausage in tomato-caper sauce	No	Not applicable
Better Homes & Gardens	April 2021	42	Coconut curry sauce	No	Not applicable
Better Homes & Gardens	April 2021	44	Coconut curry sauce: cauliflower & chickpea coconut curry	No	Not applicable
Better Homes & Gardens	May 2021	45	Warm kale-and-potato chicken salad with bacon and mustard	No	Not applicable
Better Homes & Gardens	May 2021	46	Sesame-soy noodles with shrimp	No	Not applicable
Better Homes & Gardens	May 2021	47	Zucchini-corn soup with andouille	No	Not applicable
Better Homes & Gardens	May 2021	48	Honey-garlic grilled chicken with pickled chiles & peaches	No	Not applicable
Better Homes & Gardens	May 2021	49	Skirt steak with pineapple salsa	No	Not applicable
Better Homes & Gardens	May 2021	50	Caramelized green beans	No	Not applicable
Better Homes & Gardens	May 2021	52	Not your church lady's strawberry semifreddo	No	Not applicable
Better Homes & Gardens	May 2021	53	Southern sweet tea	No	Not applicable
Better Homes & Gardens	May 2021	54	Little green dress	No	Not applicable
Better Homes & Gardens	May 2021	55	Gas station biscuits	No	Not applicable
Better Homes & Gardens	June 2021	56	Chocolate-cherry muffins	No	Not applicable
Better Homes & Gardens	June 2021	57	Sweet and spicy mango-sauced ribs with smoky slaw	No	Not applicable
Better Homes & Gardens	June 2021	58	Passion fruit shrimp & scallop skewers	No	Not applicable
Better Homes & Gardens	June 2021	59	Passion fruit shrimp & scallop skewers: fresh fruit salad skewers	No	Not applicable

Better Homes & Gardens	June 2021	60	Grilled bananas and pineapple with coconut-caramel sauce	No	Not applicable
Better Homes & Gardens	June 2021	61	Tamarind BBQ chicken	No	Not applicable
Better Homes & Gardens	June 2021	62	Coconut rice	No	Not applicable
Better Homes & Gardens	June 2021	63	Grapefruit-guava fruit punch	No	Not applicable
Better Homes & Gardens	June 2021	64	Zucchini, summer squash & chicken salad	No	Not applicable
Better Homes & Gardens	June 2021	65	Julienned snow peas, celery & radish salad	No	Not applicable
Better Homes & Gardens	June 2021	66	Thai-inspired beef & cucumber salad	No	Not applicable
Better Homes & Gardens	June 2021	67	Shaved fennel & fingerling potato salad	No	Not applicable
Better Homes & Gardens	June 2021	68	Melon, peach & prosciutto salad	No	Not applicable
Better Homes & Gardens	July 2021	70	Rainbow fruit pops	No	Not applicable
Better Homes & Gardens	July 2021	71	Affogato pops	No	Not applicable
Better Homes & Gardens	July 2021	72	Chile mango pops	No	Not applicable
Better Homes & Gardens	July 2021	73	Chocolate-toffee squares	No	Not applicable
Better Homes & Gardens	July 2021	75	Berry-yogurt rocket pops	No	Not applicable
Better Homes & Gardens	July 2021	76	Strawberry coffee cake with cornmeal streusel	No	Not applicable
Better Homes & Gardens	July 2021	77	Fresh corn salad with stone fruit & herbs	No	Not applicable
Better Homes & Gardens	July 2021	78	Corn crostata with tomatillos & queso fresco	No	Not applicable
Better Homes & Gardens	July 2021	79	Smoky grilled corn, poblano & cheese dip	No	Not applicable
Better Homes & Gardens	July 2021	80	Summer risotto with corn butter & shrimp	No	Not applicable
Better Homes & Gardens	July 2021	81	Butter-braised corn on the cob	No	Not applicable

Better Homes & Gardens	July 2021	82	Sweet corn panna cotta with cherry-vanilla compote	No	Not applicable
Better Homes & Gardens	July 2021	83	Mini cupcakes	No	Not applicable
Better Homes & Gardens	July 2021	85	Sheet-pan flag nachos	No	Not applicable
Better Homes & Gardens	August 2021	86	Green tea float	No	Not applicable
Better Homes & Gardens	August 2021	87	Herbed Arnold Palmer	No	Not applicable
Better Homes & Gardens	August 2021	88	London Fog spritz	No	Not applicable
Better Homes & Gardens	August 2021	89	London Fog spritz: vanilla syrup	No	Not applicable
Better Homes & Gardens	August 2021	90	Grandma-style pizza with capocollo & fennel	No	Not applicable
Better Homes & Gardens	August 2021	92	Chicken cashew nut	No	Not applicable
Better Homes & Gardens	August 2021	93	Roasted chile powder	No	Not applicable
Better Homes & Gardens	August 2021	94	Luna's broccoli beef	No	Not applicable
Better Homes & Gardens	August 2021	95	Sticky caramelized shrimp lettuce wraps	No	Not applicable
Better Homes & Gardens	August 2021	96	Easy wok-fried greens	No	Not applicable
Better Homes & Gardens	August 2021	97	Pad mama	No	Not applicable
Better Homes & Gardens	August 2021	98	Chicken & waffles cobb salad	No	Not applicable
Better Homes & Gardens	August 2021	99	Chicken & waffles cobb salad: maple vinaigrette	No	Not applicable
Better Homes & Gardens	August 2021	100	Shortcut sugar buns	No	Not applicable
Better Homes & Gardens	August 2021	101	Watermelon cucumber-lime refresher	No	Not applicable
Better Homes & Gardens	August 2021	102	Sparkling iced mocha	No	Not applicable
Better Homes & Gardens	August 2021	103	Pineapple-mint coconut cooler	No	Not applicable
Better Homes & Gardens	August 2021	104	Strawberry kombucha crush	No	Not applicable

Better Homes & Gardens	September 2021	109	Chocolate-hazelnut & berry folded crepe	No	Not applicable
Better Homes & Gardens	September 2021	112	Chicken laab cabbage rolls	No	Not applicable
Better Homes & Gardens	September 2021	113	Chicken laab cabbage rolls: spicy sesame dipping sauce	No	Not applicable
Better Homes & Gardens	September 2021	114	Farro salad-stuffed avocados	No	Not applicable
Better Homes & Gardens	September 2021	115	Baked stuffed tomatoes with herbs	No	Not applicable
Better Homes & Gardens	September 2021	116	Stuffed eggplant caponata	No	Not applicable
Better Homes & Gardens	September 2021	117	Poblanos with late-summer vegetables & shrimp	No	Not applicable
Better Homes & Gardens	September 2021	118	Spicy buffalo-style chicken sandwiches	No	Not applicable
Better Homes & Gardens	September 2021	120	Crispy dill pickled chicken sandwiches: copycat Chick-Fil-A sauce	No	Not applicable
Better Homes & Gardens	September 2021	121	Air-fryer chicken katsu sandwiches	No	Not applicable
Better Homes & Gardens	September 2021	124	Trailer mix energy balls	No	Not applicable
Taste of Home	February/March 2021	313	Waffle-iron pizzas	No	Not applicable
Taste of Home	February/March 2021	317	Parisian sipping chocolate	No	Not applicable
Better Homes & Gardens	October 2020	523	Moroccan chicken & cauliflower stew	No	Not applicable
Better Homes & Gardens	October 2020	524	Pork & squash stew with ginger-red cabbage slaw	No	Not applicable
Better Homes & Gardens	October 2020	525	Corn & potato stew with cornmeal dumplings	No	Not applicable
Better Homes & Gardens	October 2020	526	Roasted beet chips	No	Not applicable
Better Homes & Gardens	October 2020	528	Chicken-lentil tacos	No	Not applicable
Better Homes & Gardens	October 2020	529	Dutch baby with caramelized apples	No	Not applicable

Better Homes & Gardens	October 2020	530	Butternut squash & fettuccine alfredo	No	Not applicable
Better Homes & Gardens	November 2020	531	Ginger-cranberry relish	No	Not applicable
Better Homes & Gardens	November 2020	534	Spiced apple rings with beets	No	Not applicable
Better Homes & Gardens	November 2020	535	Red cabbage & radicchio slaw	No	Not applicable
Better Homes & Gardens	November 2020	536	Bananas foster crisp	No	Not applicable
Better Homes & Gardens	November 2020	538	Orange-almond cake with cranberry curd	No	Not applicable
Better Homes & Gardens	November 2020	539	Brioche bread pudding muffins with maple caramel	No	Not applicable
Better Homes & Gardens	November 2020	540	Whiskey and cream pumpkin tart with pecan butter shortbread crust	No	Not applicable
Better Homes & Gardens	November 2020	541	Pear tarts with caramelized pastry cream	No	Not applicable
Better Homes & Gardens	November 2020	542	Sweet potato marshmallow meringue pie	No	Not applicable
Better Homes & Gardens	November 2020	544	Winter pear salad	No	Not applicable
Better Homes & Gardens	November 2020	545	Poached pears	No	Not applicable
Better Homes & Gardens	November 2020	546	Pear-rosemary potato gratin	No	Not applicable
Better Homes & Gardens	November 2020	547	Peppery pear-vanilla scones	No	Not applicable
Better Homes & Gardens	November 2020	548	Savory pears and squash with granola tumble	No	Not applicable
Better Homes & Gardens	November 2020	550	Fire-roasted tomato strata	No	Not applicable
Better Homes & Gardens	November 2020	551	2020 Glazed roasted turkey	No	Not applicable
Better Homes & Gardens	November 2020	552	2020 Glazed roasted turkey: double mustard-brown sugar glaze	No	Not applicable
Better Homes & Gardens	November 2020	553	2020 Glazed roasted turkey: hot honey-sesame glaze	No	Not applicable

Better Homes & Gardens	November 2020	556	Roasted squash: Maple-almond butter	No	Not applicable
Better Homes & Gardens	November 2020	557	Roasted squash: Ginger-sesame	No	Not applicable
Better Homes & Gardens	December 2020	562	Tamales coloraditos	No	Not applicable
Better Homes & Gardens	December 2020	563	Crispy latkes	No	Not applicable
Better Homes & Gardens	December 2020	564	Bread bowls	No	Not applicable
Better Homes & Gardens	December 2020	566	Skillet corn bread	No	Not applicable
Better Homes & Gardens	December 2020	568	Skillet corn bread: hot pepper jelly	No	Not applicable
Better Homes & Gardens	December 2020	569	Shrimp bisque	No	Not applicable
Better Homes & Gardens	December 2020	570	Triple-ginger cookies	No	Not applicable
Better Homes & Gardens	December 2020	571	Triple-ginger cookies: ginger sugar	No	Not applicable
Better Homes & Gardens	December 2020	572	Reverse-sear prime rib with soy-ginger gravy	No	Not applicable
Better Homes & Gardens	December 2020	573	Chocolate-orange sandwich cookies	No	Not applicable
Better Homes & Gardens	December 2020	574	Raspberry marbled shortbread	No	Not applicable
Better Homes & Gardens	December 2020	575	Peppermint-chocolate brookies	No	Not applicable
Better Homes & Gardens	December 2020	576	Almond sugar cookies: buttercream frosting	No	Not applicable
Better Homes & Gardens	December 2020	577	Latte spritzes	No	Not applicable
Better Homes & Gardens	December 2020	578	Latte spritzes: coffee cream icing	No	Not applicable
Better Homes & Gardens	December 2020	579	Limoncello cheesecake bars	No	Not applicable
Better Homes & Gardens	December 2020	580	Limoncello cheesecake bars: candied lemon slices	No	Not applicable
Better Homes & Gardens	December 2020	581	Citrus shimmer	No	Not applicable
Better Homes & Gardens	December 2020	582	Almond sugar cookies	No	Not applicable

Better Homes & Gardens	December 2020	584	Almond sugar cookies: royal icing	No	Not applicable
Better Homes & Gardens	December 2020	585	Gingerbread cinnamon rolls	No	Not applicable
Better Homes & Gardens	December 2020	586	Gingerbread cinnamon rolls: spiced brown butter frosting	No	Not applicable
Better Homes & Gardens	January 2021	587	Origami fish packets	No	Not applicable
Better Homes & Gardens	January 2021	588	Flounder grenobloise	No	Not applicable
Better Homes & Gardens	January 2021	589	Crispy seasoned fish & chips with green olive tartar sauce: green olive tartar sauce	No	Not applicable
Better Homes & Gardens	January 2021	590	Buttermilk caramel cake	No	Not applicable
Better Homes & Gardens	January 2021	592	Braised cabbage collards	No	Not applicable
Better Homes & Gardens	January 2021	594	Grapefruit, champagne & vodka spritz	No	Not applicable
Better Homes & Gardens	January 2021	595	Fried green tomatillos & aioli	No	Not applicable
Better Homes & Gardens	January 2021	596	Black-eyed pea succotash	No	Not applicable
Better Homes & Gardens	January 2021	597	Cheesy grits cakes	No	Not applicable
Better Homes & Gardens	January 2021	598	Brown butter chicken & rice	No	Not applicable
Better Homes & Gardens	January 2021	599	Herbed skillet pastitsio	No	Not applicable
Better Homes & Gardens	January 2021	600	Chicken pot pie soup	No	Not applicable
Better Homes & Gardens	January 2021	601	Veggie-pesto lasagna soup	No	Not applicable
Better Homes & Gardens	January 2021	602	Loaded baked potato soup	No	Not applicable
Better Homes & Gardens	February 2021	604	Turmeric-ginger tea	No	Not applicable
Better Homes & Gardens	February 2021	605	Berry-ginger green tea sipper	No	Not applicable
Better Homes & Gardens	February 2021	609	Roasted garlic-goat cheese pizza	No	Not applicable

Better Homes & Gardens	February 2021	610	Carne asada burrito bowl	No	Not applicable
Better Homes & Gardens	February 2021	611	Homemade pizza dough	No	Not applicable
Better Homes & Gardens	February 2021	612	Veggie banh mi pizza	No	Not applicable
Better Homes & Gardens	February 2021	613	Ramen noodle salad	No	Not applicable
Better Homes & Gardens	February 2021	614	Ramen noodle salad: ginger-soy vinaigrette	No	Not applicable
Better Homes & Gardens	February 2021	615	Tahini, grain & veggie bowl	No	Not applicable
Better Homes & Gardens	February 2021	616	Sour cream donut holes with raspberry sugar & glaze	No	Not applicable
Better Homes & Gardens	February 2021	617	Sour cream donut holes with raspberry sugar & glaze: raspberry glaze	No	Not applicable
Better Homes & Gardens	February 2021	618	Sour cream donut holes with raspberry sugar & glaze: raspberry sugar	No	Not applicable
Better Homes & Gardens	February 2021	619	Strawberry-cardamom turnovers	No	Not applicable
Better Homes & Gardens	February 2021	620	Mini chocolate peanut butter pies	No	Not applicable
Better Homes & Gardens	February 2021	621	Shawarma-spiced halloumi and vegetables	No	Not applicable
Better Homes & Gardens	February 2021	622	Flank Steak tagliata with arugula & parmesan	No	Not applicable
Better Homes & Gardens	February 2021	623	Spicy broccoli rabe & chickpea skillet	No	Not applicable
Better Homes & Gardens	February 2021	625	Pesto pasta with charred radicchio	No	Not applicable
Better Homes & Gardens	February 2021	626	Broiled swordfish with fennel-caper slaw	No	Not applicable
Better Homes & Gardens	February 2021	627	Cacio e pepe farinata	No	Not applicable
Better Homes & Gardens	November 2020		Poached pears: white wine poaching liquid	No	Not applicable
Better Homes & Gardens	November 2020		Poached pears: red wine-pomegranate poaching liquid	No	Not applicable

Better Homes & Gardens	November 2020		Poached pears: citrus-maple poaching liquid	No	Not applicable
Better Homes & Gardens	February 2021		Carmelized onion jam and goat cheese crostini	No	Not applicable
Taste of Home	June/July 2021	74	Strawberry shortbread squares	No	Not applicable
Taste of Home	August/September 2021	106	Snack bar: roasted nuts	No	Not applicable
Taste of Home	August/September 2021	107	Snack bar: cheese board	No	Not applicable
Taste of Home	August/September 2021	108	Snack bar: s'mores bar	No	Not applicable
Taste of Home	October/November 2020	197	Ready, set...roll!	No	Not applicable
US Weekly	25 October 2021	997	Chocolate dipped halloween pretzels	No	Not applicable
US Weekly	25 October 2021	998	The haunted graveyard	No	Not applicable
Taste of Home	February/March 2021	284	Savory roasted chicken	Yes	Onion Salt
AARP	December 2020/January 2021	916	Cream biscuits	Yes	Salt
AARP	February/March 2021	938	What's hot? Sheet pan meals: za'atar chicken, potatoes and broccoli	Yes	Salt
AARP	February/March 2021	939	What's hot? Sheet pan meals: Mediterranean halibut	Yes	Salt
AARP	February/March 2021	940	What's hot? Sheet pan meals: flank steak fajitas	Yes	Salt
AARP	April/May 2021	926	12 new uses for strawberries: quick jam	Yes	Salt
AARP	April/May 2021	929	12 new uses for strawberries: salad dressing	Yes	Salt
Cosmopolitan	May/June 2021	954	Choose your own egg adventure: brunch bowl	Yes	Salt
Cosmopolitan	May/June 2021	957	Choose your own egg adventure: French toast	Yes	Salt
Cosmopolitan	May/June 2021	959	Choose your own egg adventure: kinda carbonara	Yes	Salt

Cosmopolitan	October 2021	968	Make yourself a pot of soup already: grandma-esque chicken soup	Yes	Salt
Cosmopolitan	November 2021	977	These 3 dips will win you most popular at the Friendsgiving table: guan 'n' cranberries dip	Yes	Salt
Cosmopolitan	November 2021	978	These 3 dips will win you most popular at the Friendsgiving table: brussels and artichoke dip	Yes	Salt
Cosmopolitan	November 2021	979	These 3 dips will win you most popular at the Friendsgiving table: buttenut buffalo chicken dip	Yes	Salt
Cosmopolitan	November 2021	980	ATTN: your chicken wants you to stuff it	Yes	Salt
Cosmopolitan	November 2020	981	Thanksgiving chicken is what micro-Friendsgiving dreams are made of	Yes	Salt
Cosmopolitan	June 2020	987	DIY dipping sauces that make veggies taste like takeout: somewhat healthy spicy mayo	Yes	Salt
Cosmopolitan	June 2020	988	DIY dipping sauces that make veggies taste like takeout: basic betch pesto	Yes	Salt
Cosmopolitan	June 2020	990	DIY dipping sauces that make veggies taste like takeout: fancy honey mustard	Yes	Salt
Cosmopolitan	October 2021	969	Make yourself a pot of soup already (mexican-ish veggie soup)	Yes	Salt
Cosmopolitan	October 2021	970	Make yourself a pot of soup already (almost minestrone)	Yes	Salt
Good Housekeeping	September 2021	656	Roasted chicken & tomatoes	Yes	Salt

Good Housekeeping	September 2021	657	Fresh zucchini salad	Yes	Salt
Good Housekeeping	September 2021	658	Grilled citrus zucchini	Yes	Salt
Good Housekeeping	September 2021	659	Cheesy zucchini crisps	Yes	Salt
Good Housekeeping	September 2020	672	Fresh side	Yes	Salt
Good Housekeeping	September 2020	673	No-cook marinara	Yes	Salt
Good Housekeeping	September 2020	674	Stuffed tomatoes	Yes	Salt
Good Housekeeping	October 2020	691	Crispy pork cutlets	Yes	Salt
Good Housekeeping	October 2020	692	Roasted chili-lime drumsticks	Yes	Salt
Good Housekeeping	October 2020	693	Curry-poached salmon with peppers	Yes	Salt
Good Housekeeping	October 2020	694	Pumpkin alfredo penne	Yes	Salt
Good Housekeeping	October 2020	695	Paprika steak with lentils & spinach	Yes	Salt
Good Housekeeping	October 2020	696	Minty peas	Yes	Salt
Good Housekeeping	October 2020	697	Tomato relish	Yes	Salt
Good Housekeeping	October 2020	698	Couscous with apricot vinaigrette	Yes	Salt

Good Housekeeping	October 2020	699	Creamy green chile sauce	Yes	Salt
Good Housekeeping	October 2020	700	Scallion-ginger sauce	Yes	Salt
Good Housekeeping	November 2020	706	Kabocha squash: maple-thyme roast	Yes	Salt
Good Housekeeping	November 2020	707	Kabocha squash: slow-cooker curry	Yes	Salt
Good Housekeeping	November 2020	708	Kabocha squash: squash toasts	Yes	Salt
Good Housekeeping	November 2020	716	Orange-buttermilk chess pie	Yes	Salt
Good Housekeeping	November 2020	721	Spiced pumpkin pie	Yes	Salt
Good Housekeeping	November 2020	724	Old-fashioned pecan pie	Yes	Salt
Good Housekeeping	May 2021	732	Asparagus-leek pasta	Yes	Salt
Good Housekeeping	May 2021	758	Artichokes: to roast	Yes	Salt
Good Housekeeping	May 2021	759	Artichokes: to grill	Yes	Salt
Good Housekeeping	March 2021	765	Mint: green pea couscous	Yes	Salt
Good Housekeeping	March 2021	766	Mint: mint & walnut pesto	Yes	Salt
Good Housekeeping	July/August 2021	825	Corn: couscous salad	Yes	Salt

Good Housekeeping	July/August 2021	826	Corn: shrimp fritters	Yes	Salt
Good Housekeeping	January/February 2021	837	Rigatoni with vodka sauce	Yes	Salt
Good Housekeeping	January/February 2021	840	Crispy chicken thighs with buttermilk fennel salad	Yes	Salt
Good Housekeeping	January/February 2021	841	Cod in parchment with orange-leek couscous	Yes	Salt
Good Housekeeping	January/February 2021	844	Balsamic-glazed sirloin with farro pilaf	Yes	Salt
Good Housekeeping	January/February 2021	848	Banh Mi sandwiches	Yes	Salt
Good Housekeeping	January/February 2021	850	Tangy avocado dip	Yes	Salt
Good Housekeeping	January/February 2021	851	Yogurt sauce	Yes	Salt
Good Housekeeping	January/February 2021	854	Endive: fennel-apple salad	Yes	Salt
Good Housekeeping	January/February 2021	855	Endive: citrus relish cups	Yes	Salt
Good Housekeeping	January/February 2021	856	Endive: roasted greens with walnuts	Yes	Salt
Good Housekeeping	December 2020	865	Beef and vegetable curry	Yes	Salt
Good Housekeeping	December 2020	866	Warm roasted cauliflower and spinach salad	Yes	Salt
Good Housekeeping	December 2020	870	Lime & Aleppo pepper green beans	Yes	Salt

Good Housekeeping	December 2020	871	Instant oatmeal with cranberries and pecans	Yes	Salt
Good Housekeeping	December 2020	875	Sheet pan chicken nachos	Yes	Salt
Good Housekeeping	December 2020	879	Roasted salmon with charred lemon vinaigrette	Yes	Salt
Good Housekeeping	April 2021	907	Scallions: tomato vinaigrette	Yes	Salt
Good Housekeeping	April 2021	908	Scallions: spring herb frittata	Yes	Salt
Good Housekeeping	November 2020		Decadent chocolate-espresso pie: for pie	Yes	Salt
Good Housekeeping	July/August 2020		Tangy relish	Yes	Salt
People	26 July 2021	640	Mango kulfi pops	Yes	Salt
People	26 July 2021	641	Cacio e pepe with parmesan crisps	Yes	Salt
People	26 July 2021	642	Grilled chile-maple chicken with tomato & watermelon salad	Yes	Salt
US Weekly	30 August 2021	993	Passion-fruit basil margarita	Yes	Salt
Taste of Home	August/September 2020	125	Apple-carrot slaw with pistachios	Yes	Salt
Taste of Home	August/September 2020	127	Crispy orange chicken	Yes	Salt
Taste of Home	August/September 2020	129	Summer garden pasta with chicken sausage	Yes	Salt
Taste of Home	August/September 2020	135	Moroccan chicken thighs	Yes	Salt
Taste of Home	August/September 2020	136	Grilled jerk shrimp orzo salad	Yes	Salt
Taste of Home	August/September 2020	140	Turkey alfredo pizza	Yes	Salt
Taste of Home	August/September 2020	142	Glazed parmesan potatoes	Yes	Salt

Taste of Home	August/September 2020	143	Mashed cauliflower	Yes	Salt
Taste of Home	August/September 2020	144	Simple Harvard beets	Yes	Salt
Taste of Home	August/September 2020	145	Slow-cooker chicken parmesan	Yes	Salt
Taste of Home	August/September 2020	146	Cheddar-topped barbecue meat flour	Yes	Salt
Taste of Home	August/September 2020	148	Rustic caramel apple tart	Yes	Salt
Taste of Home	August/September 2020	149	Apple pear cake	Yes	Salt
Taste of Home	August/September 2020	151	Slow-cooker apple pudding cake	Yes	Salt
Taste of Home	August/September 2020	152	Caramel apple muffins	Yes	Salt
Taste of Home	August/September 2020	153	Apple Betty with almond cream	Yes	Salt
Taste of Home	August/September 2020	156	Pennsylvania Dutch chocolate funny cake	Yes	Salt
Taste of Home	August/September 2020	157	Homemade tortillas	Yes	Salt
Taste of Home	August/September 2020	160	Popcorn shrimp tacos with cabbage slaw	Yes	Salt
Taste of Home	August/September 2020	162	Fruity chicken salad pitas	Yes	Salt
Taste of Home	August/September 2020	164	Rosemary salmon & veggies	Yes	Salt
Taste of Home	October/November 2020	170	Brownie haunted house	Yes	Salt
Taste of Home	October/November 2020	174	Mushroom pork ragout	Yes	Salt
Taste of Home	October/November 2020	176	Ground beef Wellingtons	Yes	Salt
Taste of Home	October/November 2020	178	Irish beef stew	Yes	Salt
Taste of Home	October/November 2020	180	The best sweet potato pie	Yes	Salt
Taste of Home	October/November 2020	183	Elegant green beans	Yes	Salt
Taste of Home	October/November 2020	187	Rosemary turkey breast	Yes	Salt

Taste of Home	October/November 2020	188	Minted beet salad	Yes	Salt
Taste of Home	October/November 2020	189	Green bean bundles	Yes	Salt
Taste of Home	October/November 2020	192	French toast spirals	Yes	Salt
Taste of Home	October/November 2020	193	Maple bacon walnut coffe cake	Yes	Salt
Taste of Home	October/November 2020	194	Maple pecan pie	Yes	Salt
Taste of Home	October/November 2020	196	Cristen's giant cinnamon rolls	Yes	Salt
Taste of Home	October/November 2020	209	Classic butter pie crust dough	Yes	Salt
Taste of Home	October/November 2020	210	Chicken with pumpkin alfredo	Yes	Salt
Taste of Home	October/November 2020	211	Pumpkin & chicken sausage hash	Yes	Salt
Taste of Home	October/November 2020	212	Pumpkin clam chowder	Yes	Salt
Taste of Home	October/November 2020	213	Ganache-topped pumpkin tart	Yes	Salt
Taste of Home	October/November 2020	214	Snickerdoodle pumpkin bread	Yes	Salt
Taste of Home	October/November 2020	215	Pumpkin rugelach with cream cheese icing	Yes	Salt
Taste of Home	October/November 2020	216	Chipotle pumpkin chicken pizza	Yes	Salt
Taste of Home	October/November 2020	219	Mini corn dogs	Yes	Salt
Taste of Home	October/November 2020	221	Spicy oatmeal cookie mix	Yes	Salt
Taste of Home	December/January 2021	226	Portuguese shrimp	Yes	Salt
Taste of Home	December/January 2021	228	Chocolate-hazelnut espresso cinnamon rolls	Yes	Salt
Taste of Home	December/January 2021	229	Easy cheesy cauliflower breakfast casserole	Yes	Salt
Taste of Home	December/January 2021	247	Christmas star twisted bread	Yes	Salt
Taste of Home	December/January 2021	249	Duo tater bake	Yes	Salt

Taste of Home	December/January 2021	250	Maple-gingerroot vegetables	Yes	Salt
Taste of Home	December/January 2021	251	Pumpkin egg braid	Yes	Salt
Taste of Home	December/January 2021	254	Triple-cheese broccoli puff	Yes	Salt
Taste of Home	December/January 2021	257	Fruitcake cookies	Yes	Salt
Taste of Home	December/January 2021	258	Mocha-walnut macarons	Yes	Salt
Taste of Home	December/January 2021	261	Roasted brussels sprouts with sriracha aioli	Yes	Salt
Taste of Home	December/January 2021	263	Cauliflower au gratin	Yes	Salt
Taste of Home	December/January 2021	265	Smoked macaroni & cheese	Yes	Salt
Taste of Home	December/January 2021	268	Tropical sweet potato bake	Yes	Salt
Taste of Home	December/January 2021	269	Jalapeno sausage quiche	Yes	Salt
Taste of Home	December/January 2021	271	Mama's coconut pie	Yes	Salt
Taste of Home	December/January 2021	272	Pineapple salsa	Yes	Salt
Taste of Home	December/January 2021	277	Potato latke funnel cakes	Yes	Salt
Taste of Home	February/March 2021	278	Chimichangas	Yes	Salt
Taste of Home	February/March 2021	280	Cinnamon toasted almonds	Yes	Salt
Taste of Home	February/March 2021	286	Spinach chicken frittata	Yes	Salt
Taste of Home	February/March 2021	288	Cranberry Nutella sandwich cookies	Yes	Salt
Taste of Home	February/March 2021	289	Brownie kiss cupcakes	Yes	Salt
Taste of Home	February/March 2021	293	Tabouleh	Yes	Salt
Taste of Home	February/March 2021	308	Beer & bacon macaroni & cheese	Yes	Salt
Taste of Home	February/March 2021	311	Crispy coconut chicken nuggets with creamy Carribbean salsa	Yes	Salt

Taste of Home	February/March 2021	312	The best French toast	Yes	Salt
Taste of Home	February/March 2021	314	Feta-stuffed kibbeh with harissa	Yes	Salt
Taste of Home	February/March 2021	315	Ceylon chicken curry & rice noodle soup	Yes	Salt
Taste of Home	February/March 2021	316	Finnish cheese pie	Yes	Salt
Taste of Home	February/March 2021	318	Conchas (seashells)	Yes	Salt
Taste of Home	February/March 2021	319	Peachy Danish pancake balls (Æbleskiver)	Yes	Salt
Taste of Home	February/March 2021	321	Vegan butter cauliflower	Yes	Salt
Taste of Home	February/March 2021	322	Indonesian bananas foster	Yes	Salt
Taste of Home	February/March 2021	326	Whiskey barbecue pork	Yes	Salt
Taste of Home	February/March 2021	328	Romesco sauce	Yes	Salt
Taste of Home	February/March 2021	329	Ash's sweet & spicy enchilada sauce	Yes	Salt
Taste of Home	April/May 2021	330	Air-fryer Nashville hot chicken	Yes	Salt
Taste of Home	April/May 2021	334	Rustic fish chowder	Yes	Salt
Taste of Home	April/May 2021	335	Alfredo sauce	Yes	Salt
Taste of Home	April/May 2021	336	Alfredo sauce: Chicken alfredo sandwiches	Yes	Salt
Taste of Home	April/May 2021	339	Homemade marinara sauce	Yes	Salt
Taste of Home	April/May 2021	340	Homemade marinara sauce: Meatball subs	Yes	Salt
Taste of Home	April/May 2021	341	Homemade marinara sauce: Italian chicken stew	Yes	Salt
Taste of Home	April/May 2021	343	Classic pesto	Yes	Salt
Taste of Home	April/May 2021	345	Classic pesto: BLT bruschetta	Yes	Salt
Taste of Home	April/May 2021	346	Classic pesto: Pesto hamburgers	Yes	Salt

Taste of Home	April/May 2021	348	Homemade biscuits & maple sausage gravy	Yes	Salt
Taste of Home	April/May 2021	349	Collards quiche	Yes	Salt
Taste of Home	April/May 2021	350	Grits & sausage casserole	Yes	Salt
Taste of Home	April/May 2021	351	Best strawberry shortcake	Yes	Salt
Taste of Home	April/May 2021	352	Bacon hash brown bake	Yes	Salt
Taste of Home	April/May 2021	370	Best deviled eggs	Yes	Salt
Taste of Home	April/May 2021	372	Gluten-free brownie bars	Yes	Salt
Taste of Home	April/May 2021	375	Garlic mozzarella bread bites	Yes	Salt
Taste of Home	April/May 2021	377	Red lentil hummus with brussels sprout hash	Yes	Salt
Taste of Home	April/May 2021	380	Gluten-free spiced sweet potato muffins	Yes	Salt
Taste of Home	April/May 2021	381	The best cashew cheese sauce	Yes	Salt
Taste of Home	April/May 2021	385	Chewy cookies	Yes	Salt
Taste of Home	June/July 2021	388	Almost It's-It ice cream sandwiches	Yes	Salt
Taste of Home	June/July 2021	391	Shrimp & scallops tropical salad	Yes	Salt
Taste of Home	June/July 2021	393	Dad's lemony grilled chicken	Yes	Salt
Taste of Home	June/July 2021	401	Skewered lamb with blackberry-balsamic glaze	Yes	Salt
Taste of Home	June/July 2021	405	Tomato galette with basil pesto & feta	Yes	Salt
Taste of Home	June/July 2021	406	Stir-fried zucchini	Yes	Salt
Taste of Home	June/July 2021	408	Baked chicken & zucchini	Yes	Salt
Taste of Home	June/July 2021	409	Summer zucchini pasta	Yes	Salt
Taste of Home	June/July 2021	410	Chocolate zucchini cookies	Yes	Salt

Taste of Home	June/July 2021	411	Carrot zucchini bread	Yes	Salt
Taste of Home	June/July 2021	412	Green tomato salsa	Yes	Salt
Taste of Home	June/July 2021	415	Fiesta corn	Yes	Salt
Taste of Home	June/July 2021	417	Blue-ribbon beef nachos	Yes	Salt
Taste of Home	June/July 2021	432	Gobi aloo	Yes	Salt
Taste of Home	June/July 2021	433	Samosas	Yes	Salt
Taste of Home	June/July 2021	434	Malai kofta	Yes	Salt
Taste of Home	June/July 2021	435	Mint chutney	Yes	Salt
Taste of Home	June/July 2021	436	Best ever vanilla ice cream	Yes	Salt
Taste of Home	June/July 2021	437	Grilled romaine salad	Yes	Salt
Taste of Home	June/July 2021	439	Cherry plum slab pie with walnut streusel	Yes	Salt
Taste of Home	June/July 2021	445	Peachy-keen halloumi fritters	Yes	Salt
Taste of Home	June/July 2021	446	Summer squash pound cake	Yes	Salt
Taste of Home	June/July 2021	447	Healthy avocado pineapple muffins	Yes	Salt
Taste of Home	June/July 2021	450	Watermelon & cucumber salsa	Yes	Salt
Taste of Home	June/July 2021	453	S'mores cupcakes	Yes	Salt
Taste of Home	August/September 2021	455	My juicy Lucy	Yes	Salt
Taste of Home	August/September 2021	459	Black bean rice burgers	Yes	Salt
Taste of Home	August/September 2021	461	Southwestern pork & squash soup	Yes	Salt
Taste of Home	August/September 2021	463	Garden chicken cacciatore	Yes	Salt
Taste of Home	August/September 2021	465	Mexican street corn chowder	Yes	Salt

Taste of Home	August/September 2021	466	Salmon grilled in foil	Yes	Salt
Taste of Home	August/September 2021	467	Sausage potato skillet	Yes	Salt
Taste of Home	August/September 2021	468	Simple salsa chicken	Yes	Salt
Taste of Home	August/September 2021	471	Oatmeal breakfast bars	Yes	Salt
Taste of Home	August/September 2021	473	Muffin-tin scrambled eggs	Yes	Salt
Taste of Home	August/September 2021	474	High-octane pancakes	Yes	Salt
Taste of Home	August/September 2021	475	Freezer breakfast sandwiches	Yes	Salt
Taste of Home	August/September 2021	476	Zucchini pizza fritters	Yes	Salt
Taste of Home	August/September 2021	477	Philly cheesesteak egg rolls	Yes	Salt
Taste of Home	August/September 2021	478	Spinach puffs	Yes	Salt
Taste of Home	August/September 2021	480	Chiles rellenos croque-madame	Yes	Salt
Taste of Home	August/September 2021	482	Steak sandwiches with crispy onions	Yes	Salt
Taste of Home	August/September 2021	485	General Tso's chicken sandwich with broccoli slaw	Yes	Salt
Taste of Home	August/September 2021	486	Toasted chicken salad sandwiches	Yes	Salt
Taste of Home	August/September 2021	488	Lebanese street sandwiches	Yes	Salt
Taste of Home	August/September 2021	491	Chewy soft pretzels	Yes	Salt
Taste of Home	August/September 2021	492	Obatzda (German beer cheese dip)	Yes	Salt
Taste of Home	August/September 2021	493	Apple matchstick salad	Yes	Salt
Taste of Home	October/November 2021	495	Baked feta pasta	Yes	Salt
Taste of Home	October/November 2021	497	30-minute coq au vin	Yes	Salt
Taste of Home	October/November 2021	499	Fluffy banana pancakes	Yes	Salt

Taste of Home	October/November 2021	501	Breakfast biscuit cups	Yes	Salt
Taste of Home	October/November 2021	505	Sweet candied carrots	Yes	Salt
Taste of Home	October/November 2021	506	Seasoned roast turkey	Yes	Salt
Taste of Home	October/November 2021	507	Mashed red potatoes	Yes	Salt
Taste of Home	October/November 2021	509	Candy corn cookies	Yes	Salt
Taste of Home	October/November 2021	510	Layered peanut butter brownies	Yes	Salt
Taste of Home	October/November 2021	515	Creamy celery root & pearl onions	Yes	Salt
Taste of Home	October/November 2021	516	Oysters Rockefeller egg rolls	Yes	Salt
Taste of Home	October/November 2021	517	Butternut squash jumble	Yes	Salt
Taste of Home	October/November 2021	518	Sweet potato kale pilaf	Yes	Salt
Taste of Home	October/November 2021	520	Good witch/bad witch cake	Yes	Salt
Better Homes & Gardens	April 2021	43	Coconut curry sauce: pork coconut curry	Yes	Salt
Better Homes & Gardens	September 2021	110	Stuffed mini chaffles	Yes	Salt
Better Homes & Gardens	September 2021	111	Baked hummingbird oatmeal	Yes	Salt
Taste of Home	February/March 2021	312	The best French toast	Yes	Salt
Taste of Home	February/March 2021	314	Feta-stuffed kibbeh with harissa	Yes	Salt
Taste of Home	February/March 2021	315	Ceylon chicken curry & rice noodle soup	Yes	Salt
Taste of Home	February/March 2021	316	Finnish cheese pie	Yes	Salt
Taste of Home	February/March 2021	318	Conchas (seashells)	Yes	Salt
Taste of Home	February/March 2021	319	Peachy Danish pancake balls (Æbleskiver)	Yes	Salt
Better Homes & Gardens	November 2020	532	Mashed potatoes	Yes	Salt

Better Homes & Gardens	November 2020	549	Soufflé pancakes with maple-pear syrup	Yes	Salt
Better Homes & Gardens	January 2021	591	Crispy seasoned fish & chips with green olive tartar sauce	Yes	Salt
Better Homes & Gardens	January 2021	593	Roasted pulled pork	Yes	Salt
Better Homes & Gardens	November 2020		Sweet potato marshmallow meringue pie: marshmallow meringue	Yes	Salt
Taste of Home	June/July 2021	84	Mini cupcakes: creamy white frosting	Yes	Salt
Taste of Home	December/January 2021	230	Haute chocolate	Yes	Salt
Taste of Home	February/March 2021	324	Slow-cooked salsa	Yes	Salt
AARP	June/July 2021		My long journey to a tasty veggie dish: grilled artichokes	Yes	Sea salt
People	2 August 2021	651	Seared scallops with cucumber & tomato relish	Yes	Sea salt
US Weekly	8 November 2021		Cream cheese pound cake	Yes	Sea salt
Taste of Home	August/September 2020	166	Chili-topped cornbread waffles	Yes	Sea salt
Taste of Home	October/November 2020	195	Pumpkin pie tartlets with maple pecan crust	Yes	Sea salt
Taste of Home	December/January 2021	241	Swedish flop	Yes	Sea salt
Taste of Home	February/March 2021	282	Caribbean shrimp bowl	Yes	Sea salt
Taste of Home	April/May 2021	347	Vegan lemon poppy seed doughnuts	Yes	Sea salt
Taste of Home	April/May 2021	379	Vegan chocolate mousse	Yes	Sea salt
Better Homes & Gardens	December 2020	567	Skillet corn bread: whipped honey butter	Yes	Sea salt
US Weekly	31 May 2021	992	Bird bakery's tomato basil soup	Yes	Sea salt
Taste of Home	October/November 2020	182	Maple-sage brined turkey	Yes	Sea salt and salt
Taste of Home	August/September 2020	158	Flavorful chicken fajitas	Yes	Seasoned salt

Taste of Home	February/March 2021	310	Baked pasta puttanesca	Yes	Seasoned salt
Taste of Home	June/July 2021	413	Mexican steak fajitas	Yes	Seasoned salt
Taste of Home	August/September 2020	132	Meatball flatbread	Yes	Yes
Taste of Home	August/September 2020	133	Parmesan pork tenderloin	Yes	Yes
Taste of Home	October/November 2020	177	Swiss potato soup	Yes	Yes
